# Prenatal Exposure to Organophosphates, Paraoxonase 1, and Cognitive Development in Childhood

**DOI:** 10.1289/ehp.1003183

**Published:** 2011-04-21

**Authors:** Stephanie M. Engel, James Wetmur, Jia Chen, Chenbo Zhu, Dana Boyd Barr, Richard L. Canfield, Mary S. Wolff

**Affiliations:** 1 Department of Preventive Medicine,; 2 Department of Microbiology, and; 3 Department of Genetics and Genomic Sciences, Mount Sinai School of Medicine, New York, New York, USA; 4 School of Public Health, Emory University, Atlanta, Georgia, USA; 5 Division of Nutritional Sciences, College of Human Ecology, Cornell University, Ithaca, New York, USA

**Keywords:** environmental exposures, IQ, mental development, organophosphates, pesticides

## Abstract

Background: Prenatal exposure to organophosphate pesticides has been shown to negatively affect child neurobehavioral development. Paraoxonase 1 (PON1) is a key enzyme in the metabolism of organophosphates.

Objective: We examined the relationship between biomarkers of organophosphate exposure, PON1, and cognitive development at ages 12 and 24 months and 6–9 years.

Methods: The Mount Sinai Children’s Environmental Health Study enrolled a multiethnic prenatal population in New York City between 1998 and 2002 (*n* = 404). Third-trimester maternal urine samples were collected and analyzed for organophosphate metabolites (*n* = 360). Prenatal maternal blood was analyzed for PON1 activity and genotype. Children returned for neurodevelopment assessments ages 12 months (*n* = 200), 24 months (*n* = 276), and 6–9 (*n* = 169) years of age.

Results: Prenatal total dialkylphosphate metabolite level was associated with a decrement in mental development at 12 months among blacks and Hispanics. These associations appeared to be enhanced among children of mothers who carried the *PON1* Q192R QR/RR genotype. In later childhood, increasing prenatal total dialkyl- and dimethylphosphate metabolites were associated with decrements in perceptual reasoning in the maternal *PON1* Q192R QQ genotype, which imparts slow catalytic activity for chlorpyrifos oxon, with a monotonic trend consistent with greater decrements with increasing prenatal exposure.

Conclusion: Our findings suggest that prenatal exposure to organophosphates is negatively associated with cognitive development, particularly perceptual reasoning, with evidence of effects beginning at 12 months and continuing through early childhood. PON1 may be an important susceptibility factor for these deleterious effects.

Before 2001, residential exposure to organophosphate pesticides, including chlorpyrifos and diazinon, was common, even in an urban setting. Insecticides are used in multiunit, inner-city dwellings to control insect and rodent infestations within apartments, in common spaces, and around building exteriors. Despite the voluntary cancellation of residential use registrations of chlorpyrifos and diazinon in 2001 and 2004, respectively, between June 2005 and March 2006, 78% of randomly selected nationally representative U.S. homes had measurable levels of chlorpyrifos, and 35% had measurable levels of diazinon, suggesting ongoing residential exposure (Stout et al. 2009). Additionally, exposure to organophosphate pesticides or their residues may occur via consumption of conventionally grown fruits and vegetables (Lu et al. 2008).

Neurodevelopmental consequences of human exposure to organophosphate pesticides have been demonstrated in urban (Bouchard et al. 2010; Engel et al. 2007; Rauh et al. 2006), rural (Eskenazi et al. 2007, 2010; Young et al. 2005), and occupational settings (Handal et al. 2008; Roldan-Tapia et al. 2006), some of which specifically involve prenatal exposure (Engel et al. 2007; Eskenazi et al. 2007, 2010; Marks et al. 2010; Rauh et al. 2006; Young et al. 2005). We have previously reported that prenatal pesticide exposure was associated with smaller head circumference (Berkowitz et al. 2004) and more abnormal primitive reflexes (Engel et al. 2007), particularly among children of mothers with low paraoxonase 1 (PON1) activity. PON1 is a key enzyme in the metabolism of organophosphate pesticides (Costa et al. 1999) and has been shown to be a biomarker of susceptibility to the toxic effects of organophophate pesticides, both in animals (Costa et al. 2003) and in humans (Engel et al. 2007; Eskenazi et al. 2010; Lee et al. 2003; Nielsen et al. 2010).

We undertook an investigation of the impact of prenatal organophosphate metabolite biomarker levels in relation to cognitive development at multiple times in childhood, while considering the modifying influence of maternal and child *PON1* genotype and enzymatic activity.

## Methods

The Mount Sinai Children’s Environmental Health Cohort study is a prospective multiethnic cohort that enrolled primiparous women who presented for prenatal care with singleton pregnancies at the Mount Sinai prenatal clinic and two private practices and were subsequently delivered at Mount Sinai Hospital between May 1998 and July 2001. The target population was healthy, first-born infants with no underlying health conditions that might independently result in serious neurodevelopmental impairment. Therefore, women were considered eligible if they were primiparous with singleton pregnancies, had no underlying health conditions that might predispose them to have serious complications of pregnancy that might result in an at-risk infant, and ultimately delivered infants that were neither extremely preterm nor very low birth weight. (Berkowitz et al. 2003, 2004). Mother–infant pairs were recruited early in pregnancy (*n* = 479). In brief, subsequent to delivery, 75 women were excluded because of medical complications, very premature births (delivery before 32 completed weeks or birth weight < 1,500 g), delivery of an infant with a birth defect, inability to collect biologic specimens before birth, change of hospital or residence outside New York City, or refusal to continue to participate, leaving 404 women–infant pairs in the final cohort (Berkowitz et al. 2004).

We administered a questionnaire to participants during their third trimester of pregnancy to obtain information on environmental exposures, sociodemographic characteristics, obstetrical and medical history, and lifestyle factors. Women self-identified as white, white Hispanic, black, black Hispanic, or other. For the purpose of this analysis, white Hispanic and black Hispanics were jointly considered Hispanic. Maternal blood and urine samples were also obtained during a routine clinical visit, generally between 26 and 28 weeks of gestation. Delivery characteristics and birth outcomes, including birth weight, length, head circumference, gestational age and infant sex, were obtained from a computerized perinatal database within the Department of Obstetrics, Gynecology and Reproductive Science at Mount Sinai Hospital.

The Bayley Scales of Infant Development, 2nd edition (BSID-II), was administered at the Mount Sinai Hospital at approximately 12 (*n* = 200) and 24 months (*n* = 276). The BSID-II provides age-standardized norms of mental [Mental Development Index (MDI)] and psychomotor [Psychomotor Development Index (PDI)] development. The MDI rates the child’s cognitive ability in a number of areas, including memory, habituation, problem solving, early number concepts, generalization, classification, vocalizations, language, and social skills. The PDI rates the child’s fine and gross motor coordination. Scales are age standardized to a mean (± SD) of 100 ± 15 (Bayley 1993). Interviews and examinations were conducted in English or Spanish as required. Children were invited to return for Wechsler psychometric intelligence tests between the ages of 6 and 9 years. Children that returned before 7 years of age were administered the Wechsler Preschool and Primary Scale of Intelligence, 3rd edition (WPPSI-III), which was administered in English or Spanish by one of four examiners. Block Design, Information, Matrix Reasoning, Vocabulary, Picture Concepts, Symbol Search, Word Reasoning, and Coding subtests were completed. Composite Verbal, Performance, Processing speed, and Full-Scale IQ (FSIQ) scores were derived using age-standardized WPPSI-III norms. Children who returned between the ages of 7 and 9 years were administered the Wechsler Intelligence Scale for Children, 4th edition (WISC-IV) by one of four examiners. Children provided witnessed assent before the start of the assessment. Block Design, Similarities, Digit Span, Picture Concepts, Coding, Vocabulary, Letter-Number Sequence, Matrix Reasoning, Comprehension, and Symbol Search subtests were completed. Composite Verbal, Perceptual Reasoning, Working Memory, Processing Speed, and FSIQ scores were derived using age-standardized WISC-IV norms. Both the WPPSI-III and the WISC-IV were administered in a private room without the parent present. Mothers provided informed consent, and children ≥ 7 years of age provided verbal and witnessed assent. This study was approved by the Institutional Review Board of Mount Sinai School of Medicine.

Maternal urine samples were analyzed by the Centers for Disease Control and Prevention (CDC) for six dialkylphosphate metabolites in two batches. Laboratory and quality control methods have been reported previously (Barr et al. 2005; Bravo et al. 2004; Wolff et al. 2005). In some cases, individual dialkylphosphate metabolite levels were missing because of analytic interference. In these cases, missing dialkylphosphate metabolite levels were imputed using regression analysis to predict the missing metabolite on the basis of the other nonmissing metabolites measured for that woman within the group of correlated metabolites, as has been previously described (Engel et al. 2007). Samples below the limit of detection (LOD) were defined as LOD/_√_^–^2. Diethyl- and dimethylphosphate metabolites were summed (as micromoles per liter) to obtain total diethylphosphate metabolites (ΣDEP) and total dimethylphosphate metabolites (ΣDMP), and total dialkylphosphate levels (ΣDAP). Very dilute samples of urine with < 20 μg/dL creatinine (*n* = 26) were excluded from organophosphate metabolite analyses, in accordance with methods that have previously been applied for spot urine biomarker measures (Carrieri et al. 2001; Engel et al. 2007; Eskenazi et al. 2004; Wolff et al. 2007). Overall, approximately 97%, 89%, and 90% of the cohort had detectable levels of ΣDAP, ΣDEP, and ΣDMP metabolites, respectively (Engel et al. 2007). A random subset of maternal peripheral blood samples from the entire cohort (*n* = 194), distributed roughly equally by maternal race/ethnicity (66 blacks, 64 Hispanics, 64 whites, the number being dictated by budgetary considerations), was analyzed for polychlorinated biphenyls (PCBs) and 1,1´-dichloro-2,2´-bis(4-chlorophenyl)ethylene (DDE). PCBs were defined as the sum of congeners 118, 153, 138, and 180 (Wolff et al. 2005). Total lipids (grams per liter) were calculated by using cholesterol and triglycerides (Phillips et al. 1989) determined on the 174 plasma samples with sufficient volume. Distributions of biomarker levels have been previously reported (Engel et al. 2007; Wolff et al. 2007).

Plasma was separated from prenatal maternal peripheral blood and cord blood at delivery and was used to measure PON1 activity by phenylacetate hydrolysis (Chen et al. 2005). Using maternal and child DNA, *PON1* polymorphisms were also measured using clamp-dependent and linking emulsion allele-specific polymerase chain reaction (Chen et al. 2005). We examined interactions between prenatal organophosphate exposure (dichotomized as above and below the median exposure) and tertiles of PON1 activity in maternal prenatal peripheral blood and child cord blood. We also examined interactions between prenatal organophosphate exposure and the maternal or child *PON1* Q192R, –108C > T, and L55M polymorphisms. We closely examined interactions if the type 3 *F*-test for the product-term in Proc GLM (either the class variable representing biomarker tertiles × dichotomized genotypes or race/ethnicity, or the linear term for log_10_ organophosphate metabolites × dichotomized genotypes or race/ethnicity) was *p* < 0.20. The *PON1* Q192R polymorphism in particular has been shown to have a strong functional consequence on the relative rate of hydrolysis of certain organophophate substrates (Li et al. 2000) and has been shown to affect the catalytic efficiency for chlorpyrifos but not diazinon (Richter et al. 2009).

Data were analyzed using SAS (version 9.1; SAS Institute Inc., Cary, NC). Generalized linear models were used to analyze the relationship between biomarker levels and MDI and PDI. In total, 200 children were administered the BSID-II at approximately 12 months of age (mean ± SD, 13.1 ± 1.6 months). Children were excluded from analyses if their refusal to do a large proportion of the items on the examination influenced their overall score (12-month MDI, *n* = 1). Two children were excluded from the 12-month analysis because their parent reported a diagnosis of pervasive developmental disorder. We also excluded observations when urine creatinine was < 20 μg/dL in the maternal urine sample (*n* = 20). Of the remaining 177 eligible children who completed 12-month exams, 174 had organophosphate metabolites measured in prenatal urine.

At the 24-month BSID-II (mean ± SD, 27.4 ± 4.5 months), 276 children completed the exam. Two were excluded from the 24-month analysis because the parent reported a diagnosis of pervasive developmental disorder. Samples of urine < 20 μg/dL creatinine (*n* = 23) were also excluded. Of the remaining 251 eligible observations, 247 had organophosphate metabolites measured in prenatal urine. We could not compute the MDI scores for 10 additional children because their refusals were too extensive to accurately calculate scores; however, they were included in the PDI analyses because their scores were valid.

To maximize the sample size of our models, we conducted analyses that combined the FSIQ, Perceptual Reasoning, Verbal Comprehension, and Processing Speed composite scores from children who came for at least one of the Wechsler psychometric intelligence exams—in the text and tables we refer to this as the “combined population” (*n* = 169). For these analyses, we preferentially selected the WISC-IV composite scores and substituted the WPPSI-III composite scores if the child did not return for the later exam. We included an indicator variable to account for whether the scores derived from a WISC-IV or WPPSI-III exam. The convergent validity between the WISC-IV and WPPSI-III has been previously reported. Specifically, in psychometric analyses, the correlation between WPPSI-III and WISC-IV FSIQ scores was 0.89; WPPSI-III Verbal IQ and WISC-IV Verbal Comprehension, 0.83; and WPPSI-III Performance IQ and WISC-IV Perceptual Reasoning, 0.79 (Flanagan and Kaufman 2004). In our study, among children who returned for both exams (*n* = 103), the correlations between the composite scores on the WPPSI-III and WISC-IV were likewise very strong: FSIQ = 0.83, Verbal IQ/Verbal Comprehension = 0.84, and Performance IQ/Perceptual Reasoning = 0.78. For all models, covariates were retained if their exclusion caused more than a 10% change in the β-coefficient of the full model or if they improved the precision of the main effect estimate. The following covariates were considered as potential confounders or effect modifiers: maternal age, race/ethnicity, marital status, education, breast-feeding, child sex, alcohol, smoking, or drug use during pregnancy, maternal IQ (measured by the Peabody Picture Vocabulary test), Home Observation for Measurement of the Environment (HOME) score, season of urine collection, maternal PON1 activity or genotype, language spoken in the home, and exact age at testing. Gestational age at delivery and birth weight were not evaluated for confounding because they are potentially causal intermediates (Moreno-Banda et al. 2009; Whyatt et al. 2004; Wolff et al. 2007). All models were adjusted for examiner and urinary creatinine.

## Results

The Mount Sinai Children’s Environmental Health Center enrolled a multiethnic inner-city cohort, most of whom were black or Hispanic women (~ 80%) (Table 1). Most mothers in the cohort were < 25 years of age at enrollment. However, in some follow-up years, those who returned for follow-up assessments tended to be older women, with a disproportionately low fraction of women in the youngest category (< 20 years at enrollment) being unreachable by any of our contact methods. We found similar trends for education. Those returning for follow-up did not differ substantially from the originally enrolled cohort with respect to racial/ethnic composition. Importantly, we also found no meaningful differences with respect to breast-feeding behaviors or alcohol use during pregnancy. In general, mothers who returned for follow-up assessments tended to have been older at enrollment and to have achieved a higher level of education. Allele frequencies for *PON1* polymorphisms varied by race, as has been previously reported for our population (Chen et al. 2003). In the population of subjects included in this analysis, the frequency of the A allele (resulting in Q amino acid) was 74% among whites, 36% among blacks, and 55% among Hispanics. The frequencies of the –108T allele and 55L alleles were also differential by race. For a detailed description of allele frequencies and metabolite concentrations according to race, see Supplemental Material, Table 1 (doi:10.1289/ehp.1003183).

**Table 1:** Characteristics of the Mount Sinai Children’s Environmental Health Study, Mount Sinai Medical Center, 1998–2002 [*n* (%)].

Original enrolled cohort (*n* = 404)	Follow-up				
	Characteristic	12 months (*n* = 200)	24 months (*n* = 276)	6–9 years (*n* = 169)
Maternal age at delivery (years)								
< 20		142 (35.2)		57 (28.5)		96 (34.8)		54 (31.9)
20–24		132 (32.7)		56 (28.0)		83 (30.1)		56 (33.1)
25–29		44 (10.9)		30 (15.0)		34 (12.3)		25 (14.8)
30–34		64 (15.8)		39 (19.5)		43 (15.6)		19 (11.2)
≥ 35		22 (5.4)		18 (9.0)		20 (7.2)		15 (8.9)
Race/ethnicity								
White		86 (21.29)		57 (28.5)		63 (22.8)		31 (18.3)
Black		112 (27.72)		52 (26.0)		74 (26.8)		47 (27.8)
Hispanic		200 (49.50)		89 (44.5)		136 (49.3)		88 (52.1)
Other		6 (1.49)		2 (1.0)		3 (1.1)		3 (1.8)
Marital status								
Married		117 (29.0)		71 (35.5)		81 (29.4)		38 (22.5)
Living with baby’s father		98 (24.3)		41 (20.5)		62 (22.5)		40 (23.7)
Single		189 (46.8)		88 (44.0)		133 (48.2)		91 (53.9)
Education								
< High school		118 (29.4)		50 (25.0)		78 (28.3)		46 (27.2)
High school graduate		83 (20.7)		35 (17.5)		55 (19.9)		36 (21.3)
Some college		103 (25.6)		49 (24.5)		70 (25.4)		51 (30.2)
≥ Bachelor’s degree		98 (24.4)		66 (33.0)		73 (26.5)		36 (21.3)
Alcohol use during pregnancy		59 (14.0)		31 (16.0)		36 (13.4)		28 (16.9)
Duration of breast-feeding (months)								
< 1		140 (42.2)		74 (37.0)		122 (44.2)		73 (43.5)
1–3		71 (21.4)		45 (22.5)		56 (20.3)		36 (21.4)
≥ 4		121 (36.5)		81 (40.5)		98 (35.5)		59 (35.1)
Any organophosphate biomarker level		383 (94.8)		190 (95.0)		267 (96.7)		165 (99.0)
Maternal *PON1* Q192R								
QQ		120 (30.9)		63 (33.3)		78 (30.2)		46 (28.2)
QR		174 (44.7)		83 (43.9)		119 (46.1)		78 (47.9)
RR		95 (24.4)		43 (22.8)		61 (23.6)		39 (23.9)
Maternal paraoxonase enzyme activity (units/mL)								
2,964–9,576 (tertile 1)		130 (33.9)		69 (37.5)		91 (35.7)		57 (34.8)
9,700–11,660 (tertile 2)		123 (32.1)		51 (27.7)		81 (31.8)		43 (26.2)
11,665–20,000 (tertile 3)		130 (33.9)		64 (34.8)		83 (32.6)		64 (39.0)


At the 12-month BSID-II exam, the estimated effect of organophosphate metabolites on the MDI was strongly heterogeneous by race/ethnicity for the ΣDAP and ΣDMP metabolites (Table 2). Among nonwhites, increasing ΣDAP and ΣDMP tertiles of exposure were associated with a decrease in the MDI [log_10_ ΣDAP: β = –3.29; 95% confidence interval (CI), –5.88 to –0.70]. However, among whites, the reverse pattern emerged, with higher exposure associating with better MDI scores (log_10_ ΣDAP: β = 4.77; 95% CI, 0.69–8.86). We found similar trends in the effect estimates when we stratified by housing type (public vs. private) rather than race/ethnicity. We found no heterogeneity in ΣDEP effect estimates according to race/ethnicity, and overall, ΣDEP metabolites were not associated with the 12-month BSID-II MDI. We found no relationship between organophosphate metabolites and the PDI at 12 months overall, and no interaction with race/ethnicity for any of the metabolite groups (Table 2). At the 24-month BSID-II, effect estimates were not heterogeneous by race/ethnicity (data not shown). Consistent with the 12-month assessment, prenatal maternal ΣDAP metabolite level was inversely associated with the 24-month MDI (β = –2.08; 95% CI, –4.60 to 0.44) in multivariate adjusted models, although the effect estimates were attenuated relative to the 12-month estimates and measured with comparable precision [see Supplemental Material, Table 2 (doi:10.1289/ehp.1003183)]. The metabolites were not associated with 24-month PDI.

**Table 2:** Prenatal organophosphate biomarker levels and 12-month BSID-II MDI in the Mount Sinai Children’s Environmental Health Study.

Organophosphate biomarker	Combined race/ethnicity*a *(*n* = 149)			Black/Hispanic subjects*b *(*n* = 111)			White subjects*b *(*n* = 38)			Interaction *p*-value
	Adjusted mean	95% CI	Adjusted mean	95% CI	Adjusted mean	95% CI				
MDI														
ΣDAP														
T3		96.1		93.1 to 99.0		91.5		88.3 to 94.7		103.7		98.5 to 108.8		< 0.001
T2		95.8		92.5 to 99.1		94.4		91.2 to 97.5		95.9		90.6 to 101.3		
T1		97.0		93.7 to 100.3		96.2		92.9 to 99.4		92.0		85.4 to 98.7		
Log_10_ β		–1.00		–3.28 to 1.28		–3.29		–5.88 to –0.70		4.77		0.69 to 8.86		0.001
ΣDEP														
T3		97.5		94.3 to 100.6		95.2		91.9 to 98.6		100.6		94.6 to 106.5		0.82
T2		95.4		92.3 to 98.6		93.8		90.4 to 97.1		96.8		90.8 to 102.9		
T1		95.9		92.9 to 98.9		94.3		90.9 to 97.6		97.3		91.8 to 102.7		
Log_10_ β		0.03		–2.23 to 2.29		–0.33		–3.00 to 2.35		0.86		–3.16 to 4.87		0.62
ΣDMP														
T3		96.1		93.4 to 99.0		92.1		89.0 to 95.2		103.3		97.9 to 108.7		< 0.01
T2		96.1		92.9 to 99.3		94.2		91.0 to 97.4		97.2		91.1 to 102.6		
T1		96.8		93.5 to 100.0		96.3		93.0 to 99.5		92.2		85.6 to 98.7		
Log_10_ β		–1.12		–3.14 to 0.89		–3.35		–5.64 to –1.06		4.45		0.82 to 8.08		< 0.001
PDI														
ΣDAP														
T3		92.5		88.5 to 96.6		94.2		89.5 to 98.9		90.8		83.3 to 98.2		0.65
T2		96.6		92.1 to 101.1		97.5		93.0 to 102.1		97.0		89.2 to 104.7		
T1		95.3		90.9 to 99.8		97.7		93.1 to 102.4		90.0		80.5 to 99.6		
Log_10_ β		–0.52		–3.66 to 2.62		–1.52		–5.21 to 2.16		2.07		–3.83 to 7.96		0.31
ΣDEP														
T3		93.6		89.3 to 98.0		95.6		91.0 to 100.2		91.7		83.5 to 99.9		0.25
T2		94.5		90.1 to 98.9		95.9		91.2 to 100.6		94.4		86.0 to 102.7		
T1		95.3		91.2 to 99.5		97.7		93.1 to 102.4		92.1		84.6 to 99.6		
Log_10_ β		–0.20		–3.28 to 2.87		–0.48		–4.11 to 3.16		0.46		–5.12 to 6.03		0.78
ΣDMP														
T3		94.5		90.6 to 98.5		96.4		92.0 to 100.8		92.5		84.9 to 100.2		0.83
T2		93.7		89.3 to 98.0		94.5		90.1 to 99.0		94.4		86.7 to 102.1		
T1		95.1		90.7 to 99.5		97.8		93.2 to 102.4		89.5		80.2 to 98.8		
Log_10_ β		–0.92		–3.68 to 1.85		–1.81		–5.07 to 1.45		1.36		–3.83 to 6.56		0.31
T, tertile. **a**General linear model adjusted for race/ethnicity, maternal age at enrollment, child sex, examiner, maternal PON1 enzyme activity, season of urine collection, laboratory batch, HOME score, alcohol consumption during pregnancy, and urinary creatinine. **b**General linear model adjusted for maternal age at enrollment, child sex, examiner, maternal PON1 enzyme activity, season of urine collection, laboratory batch, HOME score, alcohol consumption during pregnancy, and urinary creatinine and including a biomarker–race interaction term.														

Because of the strong interaction between race/ethnicity and organophosphate metabolites on the BSID-II MDI at 12 months, we examined the interaction between the *PON1* polymorphisms and ΣDAP and ΣDMP metabolites within strata of race/ethnicity; however, the white population was too small to further subdivide by genotype. Therefore, we restricted this analysis to only the black or Hispanic population. Among blacks and Hispanics, the effects of ΣDAP, ΣDEP, and ΣDMP were strongly differential according to *PON1* Q192R genotype at 12 months but not 24 months. At 12 months, children of mothers with the *PON1* 192 QR/RR genotype experienced approximately a 5-point decline on the MDI with each log_10_ unit increase in ΣDAP or ΣDMP biomarker level which was monotonic across tertiles [see Supplemental Material, Figure (doi:10.1289/ehp.1003183)], and a 2-point decline in MDI for each log_10_ unit increase in ΣDEP biomarker level (Table 3). There was essentially no effect among children of mothers with the QQ genotype. Although in some instances the point estimates among the QQ group were quite elevated in a positive direction, they were always estimated with imprecision and did not follow a monotonic pattern. Results were consistent when we stratified by black and Hispanic ancestry (see Supplemental Material, Table 3), although the sample size was quite small. We found no interaction between the *PON1* Q192R polymorphism and organophosphate metabolite level on the MDI at 24 months. We also found no interactions (*p* ≥ 0.20) between organophosphate metabolites and the L55M or –108C > T polymorphisms, or with enzyme activity, on neurodevelopment at any age. Results were similar when we estimated associations according to the child’s *PON1* Q192R genotype instead of the mother’s genotype, but child genotype was available for only 57% of the population because cord blood was collected for only a subset. Therefore, we report only the maternal gene–organophosphate interactions.

**Table 3:** *PON1 *Q192R interaction with ΣDAP, ΣDEP, and ΣDMP levels on the BSID-II MDI in the Mount Sinai Children’s Environmental Health Study.

12-month BSID-II: black/Hispanic subjects (*n* = 110)*a*									24-month BSID-II: total population (*n* = 197)								
*PON1* 192 QQ (slow; *n* = 28)			*PON1* 192 QR/RR (fast;*b n* = 82)			Interaction *p*-value	*PON1* 192 QQ (slow; *n* = 57)			*PON1* 192 QR/RR (fast; *n* = 140)			Interaction *p*-value
Biomarker	log_10_ β	95% CI	log_10_ β	95% CI	log_10_ β		95% CI	log_10_ β	95% CI				
ΣDAP		5.72		–0.48 to 11.92		–4.94		–7.81 to –2.07		< 0.01		–1.04		–6.06 to 3.99		–1.27		–4.40 to 1.84		0.93
ΣDEP		3.69		–0.97 to 8.36		–1.95		–5.36 to 1.47		0.06		–0.55		–4.79 to 3.70		–0.15		–3.51 to 3.21		0.88
ΣDMP		2.76		–2.44 to 7.97		–4.47		–7.05 to –1.89		0.02		0.12		–4.17 to 4.42		–0.48		–3.27 to 2.30		0.81
Data are for a general linear model adjusted for maternal age at enrollment, child sex, examiner, HOME score, alcohol consumption during pregnancy, laboratory batch, season of urine collection, urinary creatinine, and including a biomarker–*PON1 *Q192R genotype interaction. The 24-month model was additionally adjusted for maternal race/ethnicity. **a**One subject identified mixed ethnicity. **b**In this category, seven were black and the remainder Hispanic.																				

**Figure 1:**
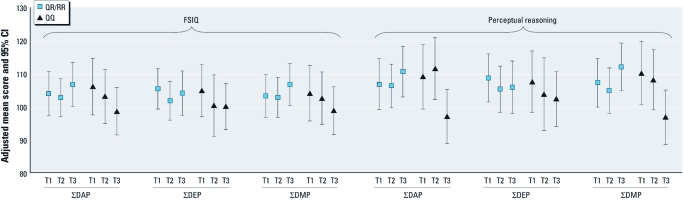
Multivariate adjusted mean estimates and 95% CIs according to tertiles (T) of exposure and *PON1* Q192R genotype. Among the children of mothers with the *PON1* 192QQ genotype (triangles), increasing tertile of ΣDAP, ΣDEP, and ΣDMP exposure was generally associated with a monotonic decline in the combined WISC-IV/WPPSI-III FSIQ and Perceptual Reasoning domains, adjusted for sex, race/ethnicity, maternal education, language in the home, alcohol use in pregnancy, batch and season of urine collection, urinary creatinine, and an indicator variable to designate the WISC-IV or WPPSI-III instrument. We found no consistent patterns in the QR/RR genotype group (squares). There was considerable imprecision in all estimates. The first- versus third-tertile contrasts for Perceptual Reasoning were significantly different at *p* < 0.05 for ΣDAP and ΣDMP.

Increasing ΣDEP metabolite levels were associated with slight decrements in FSIQ (log_10_ β = –2.89; 95% CI, –6.15 to 0.36) and Perceptual Reasoning (log_10_ β = –3.51; 95% CI, –7.31 to 0.30) on the combined psychometric exams, and also with Working Memory (log_10_ β = –3.48; 95% CI, –7.29 to 0.34) on the 7–9 year WISC-IV examination (Table 4), although the estimated effects were relatively modest and imprecise. These associations were not heterogeneous by race/ethnicity. However, for the combined population, we again found effect heterogeneity according to the *PON1* Q192R polymorphism for the associations between ΣDAP and ΣDMP biomarkers and perceptual reasoning domain. In contrast to the 12-month BSID-II MDI, children of mothers with the slow-catalytic-activity genotype (QQ) experienced a substantial decrement in the overall perceptual reasoning score with each log_10_ increment increase in exposure, whereas we found no effect of ΣDAP and ΣDMP biomarkers on the perceptual reasoning score of children with mothers who carried the QR/RR genotypes (Table 5). Comparing biomarker tertiles according to genotype, increasing tertiles of ΣDAP, ΣDEP, and ΣDMP among the children of mothers with the *PON1* 192QQ genotype were generally associated with monotonically declining adjusted means in FSIQ and perceptual reasoning, whereas we found no consistent pattern in the QR/RR group (Figure 1). For perceptual reasoning, the first- versus third-tertile contrasts for ΣDAP and ΣDMP were statistically significant (*p* < 0.05).

**Table 4:** Prenatal organophosphate biomarker levels and psychometric intelligence at 6–9 years in the Mount Sinai Children’s Environmental Health Study.

Log_10_ ΣDAP					Log_10_ ΣDEP					Log_10_ ΣDMP				
*n*	β	95% CI	*n*	β	95% CI	*n*	β	95% CI
Combined populations (6–9 years)																		
FSIQ		140		–1.39		–4.54 to 1.77		140		–2.89		–6.15 to 0.36		142		–0.46		–3.17 to 2.26
Perceptual Reasoning				–2.36		–6.04 to 1.31				–3.51		–7.31 to 0.30				–1.15		–4.31 to 2.02
Verbal Comprehension				–0.42		–3.45 to 2.62				–1.20		–4.35 to 1.96				–0.05		–2.64 to 2.54
WISC-IV (7–9 years)																		
FSIQ		114		–1.10		–5.01 to 2.81		114		–3.15		–7.19 to 0.89		115		–0.39		–3.64 to 2.86
Perceptual Reasoning				–2.39		–6.97 to 2.19				–4.37		–9.10 to 0.36				–1.24		–5.05 to 2.57
Verbal Comprehension				0.56		–3.11 to 4.23				–0.08		–3.91 to 3.76				0.39		–2.65 to 3.42
Processing Speed				–1.05		–5.57 to 3.46				–2.11		–6.81 to 2.59				–0.79		–4.52 to 2.94
Working Memory				–0.53		–4.24 to 3.18				–3.48		–7.29 to 0.34				0.29		–2.81 to 3.38
WPPSI-III (6 years)																		
FSIQ		96		–1.14		–4.55 to 2.28		96		–1.40		–5.27 to 2.47		98		–0.56		–3.68 to 2.56
Perceptual Reasoning				–2.07		–5.66 to 1.52				–1.59		–5.68 to 2.50				–1.46		–4.74 to 1.83
Verbal Comprehension				–1.16		–4.59 to 2.27				–2.27		–6.14 to 1.60				–0.52		–3.67 to 2.62
Processing Speed				–1.22		–5.12 to 2.67				–1.85		–6.25 to 2.56				–0.84		–4.35 to 2.67
Data are for a generalized linear models adjusted for sex, race/ethnicity, maternal education, language in the home, maternal PON1 enzymatic activity, alcohol use in pregnancy, batch season of urine collection, and urinary creatinine. Combined population models additionally adjusted for whether the score came from the WISC-IV or WPPSI-III instrument.																		

**Table 5:** Joint prenatal organophosphate biomarker and *PON1* Q192R effect on combined IQ domains at 6–9 years in the Mount Sinai Children’s Environmental Health Study.

*PON1* 192 QR/RR (fast; *n* = 101)			*PON1* 192 QQ (slow; *n* = 39)			Interaction *p*-value
β	95% CI	β	95% CI			
FSIQ										
Log_10_ ΣDAP		–0.66		–4.33 to 3.00		–2.33		–8.40 to 3.74		0.64
Log_10_ ΣDEP		–2.32		–6.49 to 1.86		–3.13		–8.21 to 1.96		0.80
Log_10_ ΣDMP		0.28		–2.89 to 3.44		–1.79		–6.83 to 3.25		0.49
Perceptual Reasoning										
Log_10_ ΣDAP		–0.56		–4.80 to 3.68		–7.52		–14.53 to –0.51		0.09
Log_10_ ΣDEP		–3.24		–8.11 to 1.62		–4.80		–10.73 to 1.13		0.68
Log_10_ ΣDMP		0.71		–2.96 to 4.38		–6.15		–11.99 to –0.31		0.05
Verbal Comprehension										
Log_10_ ΣDAP		–0.33		–3.87 to 3.20		0.73		–5.12 to 6.59		0.76
Log_10_ ΣDEP		–0.45		–4.51 to 3.60		–1.20		–6.13 to 3.74		0.81
Log_10_ ΣDMP		0.12		–2.93 to 3.16		0.24		–4.60 to 5.09		0.97
Generalized linear models adjusted for sex, race/ethnicity, maternal education, language in the home, alcohol use in pregnancy, batch season of urine collection, urinary creatinine, and an indicator variable to designate the WISC-IV or WPPSI-III instrument.										

Maternal third-trimester blood PCB concentration was inversely associated with mental development at 12 months (log*_e_* β = –7.18; 95% CI, –14.47 to 0.11) but not at 24 months (log*_e_* β = 1.60; 95% CI, –6.57 to 9.77) and was not associated with IQ [see Supplemental Material, Table 4 (doi:10.1289/ehp.1003183)]. However, although the effect estimates for PCBs were sometimes quite large, they were also measured with extreme imprecision, as indicated by the very large CIs. Maternal third-trimester blood DDE was not associated with any of the outcome measures.

## Discussion

We report an association between prenatal increasing ΣDAP and ΣDMP urinary metabolite concentrations and poorer scores on the BSID-II MDI at 12 months among blacks and Hispanics. At some ages, these associations were modified by maternal *PON1* Q192R genotype, although the interaction effects across exam years were not entirely consistent. For the 12-month exam, the strongest negative associations with organophosphate exposure was found in the QR/RR group, whereas for the psychometric exams the strongest negative associations were found in the QQ group. An explanation for a switch in the enhanced-risk genotype is not readily apparent; however, *PON1* is a complex gene involved in multiple physiological processes, including organophosphate metabolism but also lipid peroxidation and oxidative stress (Li et al. 2003), which may impact neurodevelopment independently (Eskenazi et al. 2010) and/or jointly with organophosphate exposure.

Heterogeneity according to maternal race/ethnicity may indicate differences in exposure sources rather than any underlying susceptibility. As previously described, overall, 46.4% of our mothers reported that pesticides were applied in their home either by themselves or by a family member during their pregnancy, although there was a profound racial disparity in this behavior (Berkowitz et al. 2003). Among the women who returned for the 12-month BSID-II exam in the present study, 70.5% of the blacks and Hispanics reported buying pesticides, applying pesticides, or fumigating their house during their pregnancy, compared with only 31.6% of the whites, and yet we did not detect significant differences in their exposure distributions [see Supplemental Material, Table 1 (doi:10.1289/ehp.1003183)]. In our population, race/ethnicity was strongly associated with whether a subject lived in public or private housing. And indeed, we found similar trends in the effect estimates when we stratified by housing type (public vs. private) rather than race/ethnicity, although the magnitude of the interaction was not as strong. This indicates that whites, or people living in private or owner-occupied housing, may have experienced a different source of exposure to pesticides or their metabolites that contributed substantially to their urinary concentrations; one possibility is that pesticide residues from fresh fruit and vegetable consumption account for a large fraction of the urinary metabolite levels among whites. Unfortunately, this presents serious complications for exposure reconstruction using urinary metabolites. A recent examination of dialkylphosphate residues on fruits and vegetables found that more than half of the samples tested contained more preformed dialkylphosphate residues than parent organophosphate pesticides (Zhang et al. 2008). These dialkylphosphate residues were produced by abiotic hydrolysis, photolysis, or plant metabolism (Zhang et al. 2008). Direct intake of the metabolite (i.e., pesticide residue) without the active oxon, rather than the parent pesticide, does not inhibit cholinesterase activity. Thus, for subjects for whom the primary source of pesticide exposure is fresh fruit and vegetable consumption, use of urinary metabolite concentrations as an indication of parent compound exposure may result in significant misclassification of exposure.

Prenatal ΣDEP urinary metabolite concentrations were associated with slight decrements in FSIQ, Perceptual Reasoning, and Working Memory between the ages of 6 and 9 years. Furthermore, among children of QQ mothers, ΣDAP and ΣDMP urinary metabolite concentrations were associated with poorer scores on Perceptual Reasoning and FSIQ in a monotonically decreasing manner. In both cases, we observed stronger Q192R interactions for ΣDAP and ΣDMP urinary metabolites, rather than ΣDEP metabolites. The reasons for this are unclear. *PON1* Q192R exhibits substrate specificity (Richter et al. 2009), but our strongest interactions were for dimethylphosphates, not diethylphosphates (into which chlorpyrifos and diazinon both metabolize). An alternative explanation is that our dimethylphosphate metabolite concentrations were simply higher (Engel et al. 2007), indicating more available substrate. Unfortunately, the relevant parent compounds cannot be deduced on the basis of nonspecific urinary dialkylphosphates; however, one well-publicized source of dimethylphosphate exposure during the period of enrollment was malathion spraying for mosquitoes carrying West Nile virus (Nash et al. 2001; O’Sullivan et al. 2005). Interestingly, *PON1* status may indirectly influence methyl organophosphate metabolism when multiple organophosphate exposures are involved (Jansen et al. 2009).

The estimated effects we report for the BSID-II MDI are in line with what has recently been reported in the CHAMACOS (Center for the Health Assessment of Mothers and Children of Salinas) study (Eskenazi et al. 2010), a similarly designed prospective birth cohort that enrolled members of an agricultural community in the Salinas Valley of California. Eskenazi et al. (2010) reported evidence of negative effects of ΣDAPs on the MDI, but not the PDI, and particularly among children with the QQ genotype, although there did not appear to be strong effect heterogeneity in the dialkylphosphate–MDI relationship according to *PON* genotype, and in some circumstances the RR and QQ effect estimates were extremely similar. Although we report interactions with maternal *PON1* Q192R genotype, we found very similar interactions and effect estimates for child genotype in our cohort (data not shown). In no cases did we detect effect heterogeneity according to PON1 enzyme activity, perhaps because genotype is a more stable, long-term predictor of metabolism potential. Reconciling estimated effects across studies can be complicated when only nonspecific urinary metabolites are measured because these metabolites can derive from multiple parent compounds (CDC 2010) that may vary in the degree to which they interact with the Q192R genotype and influence neurodevelopment.

Ours was a multiethnic study population recruited at an inner-city tertiary care hospital that serves a lower-income minority population. Thus, attrition resulting from the challenges of maintaining contact with this population may have affected our study findings. Misclassification of parent compound exposure according to exposure route is another significant limitation of our study and other studies relying on urinary dialkylphosphate biomarkers and may explain the heterogeneity in effects observed according to race/ethnicity in our population. It may further complicate the comparison of exposure effects across studies where heterogeneity exists in exposure sources. Additionally, we measured dialkylphosphate biomarkers at one time during pregnancy, approximately the early third trimester. Although we accounted for seasonal variation in exposure levels in our model, other time-related variability may result in additional misclassification of exposure.

Finally, residual confounding by unmeasured covariates, including postnatal exposure, should be considered when evaluating our results. Our study enrollment and evaluation periods overlapped with important regulatory changes in residential use of chlorpyrifos and diazinon, which occurred approximately midway through our study. Therefore, childhood exposure to these compounds may vary substantially pre- versus postban, although we did not observe any significant interaction with study year (data not shown). However, these changes may explain the inconsistency between our 12- and 24-month BSID-II results. The 0- to 12-month exposure window may include less hand-to-mouth childhood exposure (from crawling exposure to house dust, and/or fresh fruit and vegetable consumption) than in the 12- to 24-month window; thus, more childhood exposure in the 12- to 24-month window may modify the effect of prenatal exposure. Nonetheless, it is likely, overall, that childhood exposure to chlorpyrifos and diazinon was lower postban (Williams et al. 2008).

## Conclusion

We found that prenatal maternal urinary dialkylphosphate metabolite concentrations were negatively associated with aspects of neurodevelopment at 12 and 24 months, and also at 6–9 years of age, in an urban, inner-city population. At the later ages, the evidence was strongest among the children of mothers with the *PON1* 192QQ genotype, which was present in approximately 30% of our population overall, although that varies according to racial ancestry. This important potential source of effect heterogeneity should be considered in future studies of organophosphate exposure.

## Correction

Estimated betas and confidence intervals for the 12 and 24 month MDI in Table 3 and Supplemental Material Table 3 were inadvertently attributed to the incorrect genotype in the manuscript originally published online. Estimated betas and confidence intervals previously ascribed to the QQ genotype instead belonged to the QR/RR genotype, and vice versa. Therefore the negative of exposure on MDI at 12 months were actually found among the QR/RR group, with essentially no effect found in the slow-catalytic activity QQ genotype. A revised Supplemental Material file has been placed online.

## Supplemental Material

(124 KB) PDF
