# Supplementary material for: Prenatal Exposure to Organophosphates, Paraoxonase 1, and Cognitive Development in Childhood
Source: Environ Health Perspect. 2011 Apr 21;119(8):1182–8. doi: 10.1289/ehp.1003183 (PMC3237356; doi:10.1289/ehp.1003183)
Supplement: (124 KB) PDF [file ehp.1003183.s001.pdf]

Supplemental Material

Prenatal Exposure to Organophosphates, Paraoxonase 1, and Cognitive Development in Childhood

Stephanie M Engel, James Wetmur, Jia Chen, Chenbo Zhu, Dana Boyd Barr, Richard L. Canfield, and , Mary S Wolff

Supplemental Material, Table 1 PON Genotype and Phenotype Distributions and Dialkylphosphate Metabolite distributions according to race/ethnicity for subjects returning for at least 1 postnatal neurodevelopmental exam between 12 months and 9 years of age (n = 318)

	Total Population (n = 318)[a]		Whites (n =71)		Blacks (n = 88)		Hispanics (n = 156)	
	N	%	N	%	N	%	N	%
**PON Q192R [b]**	308		67		86		152	
**QQ**	92	30%	38	57%	13	15%	40	26%
**QR**	143	46%	25	37%	41	48%	76	50%
**RR**	73	24%	4	6%	32	37%	36	24%
**PON -108C>T [b]**	**310**		**67**		**88**		**152**	
**TT**	48	15%	26	39%	2	2%	19	13%
**CT**	120	39%	29	43%	22	25%	68	45%
**CC**	142	46%	12	18%	64	73%	65	43%
**PON L55M [b]**	310		67		88		152	
**LL**	27	9%	14	21%	3	3%	9	6%
**LM**	130	42%	32	48%	32	36%	66	43%
**MM**	153	49%	21	31%	53	60%	77	51%
**PON Enzymatic Activity (units/mL)**	304		64		84		153	
**Tertile 1 (2964 - 9576)**	108	36%	22	34%	35	42%	50	33%
**Tertile 2 (9700 - 11660)**	95	31%	19	30%	27	32%	48	31%
**Tertile 3 (11665 - 19674)**	101	33%	23	36%	22	26%	55	36%
**∑ DAP (nm/g Creatinine) [b]**	294		62		81		148	
**Tertile 1 (0.002 - 0.57)**	96	33%	8	13%	29	36%	57	39%
**Tertile 2 (0.57 - 1.79)**	94	32%	25	40%	20	25%	49	33%
**Tertile 3 (1.79 - 85.79)**	104	35%	29	47%	32	40%	42	28%
**∑ DEP (nm/g Creatinine)**	294		62		81		148	
**Tertile 1 (0.002 - 0.15)**	96	33%	16	26%	29	36%	49	33%
**Tertile 2 (0.15 - 0.50)**	94	32%	19	31%	25	31%	49	33%
**Tertile 3 (0.50 - 16.43)**	104	35%	27	44%	27	33%	50	34%
**∑ DMP (nm/g Creatinine) [b]**	304		64		84		153	
**Tertile 1 (0.002 - 0.30)**	97	32%	10	16%	28	33%	57	37%
**Tertile 2 (0.31 - 1.17)**	100	33%	25	39%	26	31%	49	32%
**Tertile 3 (1.18 - 85.69)**	107	35%	29	45%	30	36%	47	31%

	Median	IQR (25%, 75%)	% detect	Median	IQR	% detect	Median	IQR	% detect	Median	IQR	% detect
**∑ DAP (nm/L)**	81.3	31.7, 198.1	97.3	88.1	31.7, 192.9	98.4	81.5	42.9, 243.7	96.3	76.2	28.0, 176.7	97.3
**∑ DEP (nm/L)**	20.2	7.8, 54.6	87.8	19.9	7.1, 42.2	87.1	16.9	8.0, 64.2	85.2	21.0	8.1, 58.0	89.2
**∑ DMP (nm/L)**	44.8	16.0, 149.4	96.4	55.0	22.0, 165.1	96.9	47.8	19.1, 182.9	95.2	37.0	13.1, 125.4	96.7

[a] 3 people self-identified as not black, white or hispanic; [b] Significant difference in race-specific proportions or means according to chi-square or Kruskal-Wallis test as appropriate, at $p < 0.05$.

Supplemental Material, Table 2 Prenatal Organophosphate Biomarker Levels and 24-month BSID-II Mental and Psychomotor Development Index in the Mount Sinai Children's Environmental Health Study

		Mental Development Index N = 208		Psychomotor Development Index N = 210	
		**Adjusted Mean MDI (Beta)**	**95% CI**	**Adjusted Mean PDI (Beta)**	**95% CI**
	**T3**	90.3	85.9, 94.7	95.1	90.9, 99.2
**ΣDAP**	**T2**	90.8	86.3, 95.3	94.5	90.2, 98.8
	**T1**	93.6	89.1, 98.0	94.8	90.5, 99.1
	**$\log_{10}$**	(-2.08)	(-4.60, 0.44)	(0.93)	(-1.41, 3.28)
	**T3**	91.2	86.6, 95.7	94.8	90.5, 99.1
**ΣDEP**	**T2**	90.5	86.1, 94.9	94.9	90.6, 99.1
	**T1**	92.6	88.2, 97.0	94.7	90.5, 98.9
	**$\log_{10}$**	(-1.47)	(-3.99, 1.04)	(0.67)	(-1.72, 3.06)
	**T3**	91.1	86.9, 95.3	94.2	90.2, 98.1
**ΣDMP**	**T2**	92.9	88.6, 97.1	95.4	91.4, 99.5
	**T1**	92.5	88.0, 96.9	94.8	90.5, 99.0
	**$\log_{10}$**	(-0.93)	(-3.11, 1.25)	(0.36)	(-1.70, 2.43)

† General linear model adjusted for race/ethnicity, maternal age at enrollment, child sex, examiner, maternal education, maternal PON1 enzyme activity, season of urine collection, laboratory batch, HOME Score, alcohol consumption during pregnancy, and urinary creatinine.

Supplemental Material, Table 3 *PON1* Q192R Interaction with Total Dialkyl and Dimethylphosphate Biomarker levels on the 12-Month BISD-II Mental Development Index in the Mount Sinai Children's Environmental Health Study Among Blacks and Hispanics

	**12-MONTH BSID-II AMONG ONLY BLACKS N = 44**					**12-MONTH BSID-II AMONG ONLY HISPANICS N = 66**				
	**QQ (SLOW) N = 7**		**QR/RR (FAST) N = 37**			**QQ (SLOW) N = 23**		**QR/RR (FAST) N = 43**		
**Organophosphate Metabolites**	**Beta**	**95% CI**	**Beta**	**95% CI**	**Interaction p-value**	**Beta**	**95% CI**	**Beta**	**95% CI**	**Interaction p-value**
**$\log_{10}$ ΣDAP**	18.75	4.63, 32.88	-4.23	-9.15, 0.70	<0.01	-1.83	-10.39, 6.74	-4.63	-8.70, -0.57	0.56
**$\log_{10}$ ΣDEP**	20.56	1.77, 39.36	-0.09	-6.53, 6.35	0.04	0.22	-5.17, 5.61	-2.35	-6.99, 2.30	0.39
**$\log_{10}$ ΣDMP**	15.43	2.14, 28.72	-3.98	-7.97, 0.01	<0.01	-3.22	-9.58, 3.14	-4.57	-8.45, -0.69	0.72

General linear model adjusted for maternal age at enrollment, child sex, examiner, HOME Score, alcohol consumption during pregnancy, laboratory batch, season of urine collection, urinary creatinine, and including a biomarker-*PON1* Q192R genotype interaction. The 24-month model was additionally adjusted for maternal race/ethnicity.

Supplemental Material, Table 4 Prenatal PCB and DDE Biomarker Levels and Neurodevelopment in the Mount Sinai Children's Environmental Health Study

**Assessment**			**Log_e PCB**			**Log_e DDE**	
		**N**	**Beta**	**95% CI**	**N**	**Beta**	**95% CI**
**MDI** [a]	**12 Month**	87	-7.18	-14.47, 0.11	87	-1.87	-6.45, 2.72
	**24 Month**	102	1.60	-6.57, 9.77	102	-2.34	-7.18, 2.51
**PDI** [a]	**12 Month**	86	-3.57	-14.85, 7.71	86	2.03	-4.89, 8.95
	**24 Month**	104	2.97	-5.55, 11.49	104	-0.48	-5.23, 4.28
**Combined** [b] **WISC-WPPSI**	**FSIQ**	64	4.25	-8.88, 17.38	64	4.46	-6.62, 15.54
	**Perceptual Reasoning**	64	-0.33	-14.38, 13.72	64	2.02	-9.84, 13.89
	**Verbal Comprehension**	64	4.52	-9.84, 18.89	64	1.66	-10.52, 13.84

[a] General linear model adjusted for race/ethnicity, maternal age at enrollment, child sex, examiner, maternal education, HOME Score, alcohol consumption during pregnancy, and blood lipids.

[b] Generalized linear models adjusted for sex, race, maternal education, language in the home, alcohol use in pregnancy, blood lipids, and a test indicator variable.

Supplemental Figure. BSID-II MDI at 12 months according to summed dialkylphosphate metabolite tertiles and *PON1* Q192R genotype

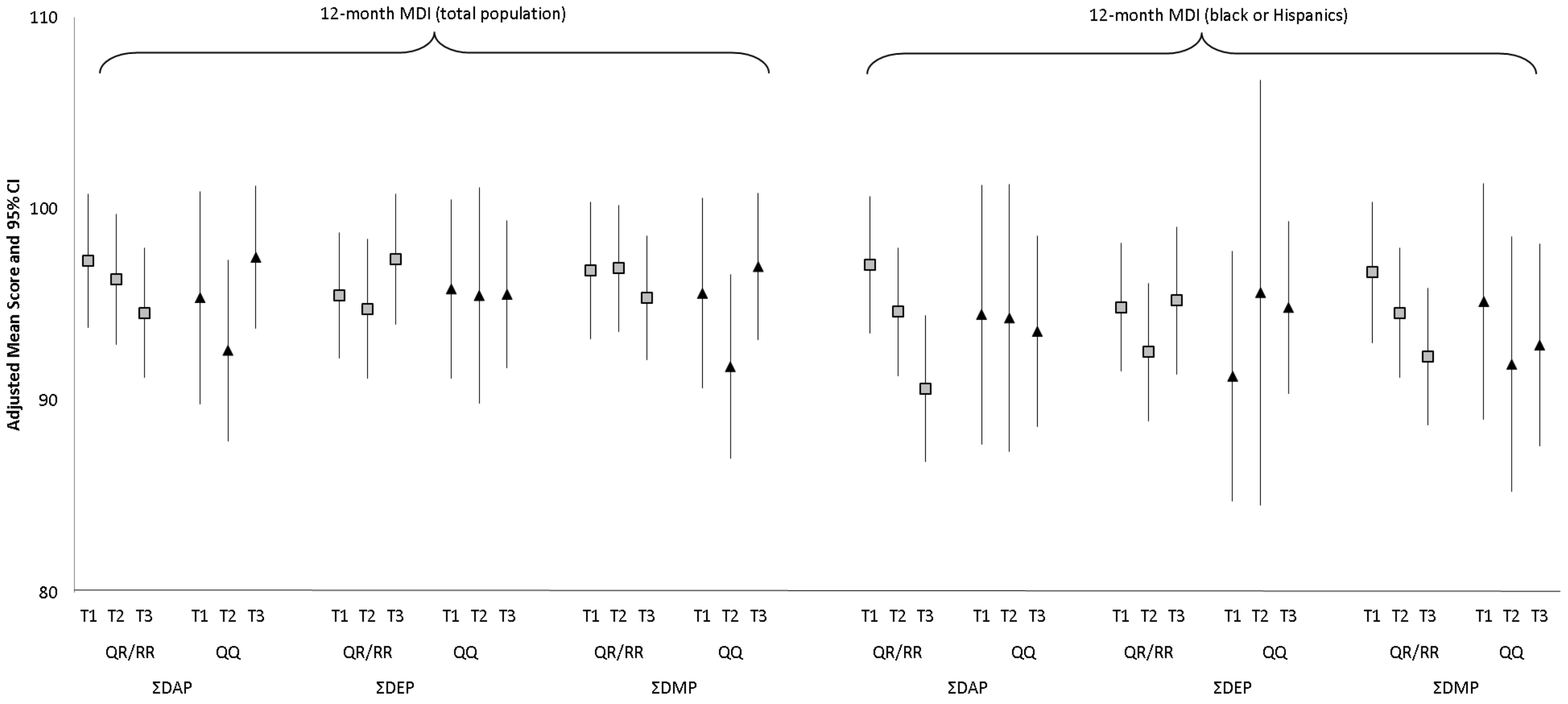

Supplemental Figure. Multivariate adjusted mean estimates and 95% CIs according to tertiles of exposure and *PON1* Q192R genotype. Among the children of mothers with the *PON1* 192QR/RR genotype (squares), increasing tertile (T) of ΣDAP and ΣDMP exposure was generally associated with a monotonic decline in the 12-month MDI among blacks and Hispanics, and also in the total population, adjusted race/ethnicity, maternal age at enrollment, child sex, examiner, maternal PON1 enzyme activity, season of urine collection, laboratory batch, HOME score, alcohol consumption during pregnancy, and urinary creatinine. We found no consistent patterns in the QQ genotype group (triangles). There was considerable imprecision in all estimates.
